# Items of General Interest

**Published:** 1916-05

**Authors:** 


### Items of General Interest.

Dr. A. A. Nelson, of Nacogdoches, is having installed in his offices a large new X-ray machine.

The Northwest Texas Medical Association held its regular session in Seymour, April 11th and 12th.

The 1916 meeting of the American Academy of Medicine will be held in Detroit, Mich., June 9th to 12th.

Dr. C. M. Cash, former city-county health officer of Abilene, was elected mayor of San Benito, Cameron county, a few days ago.

The eighteenth annual meeting of the American Proctologic Society will be held in Hotel Statler, Detroit, Mich., June 12 and 13, 1916.

More than one thousand doctors are expected to attend the annual convention of the Texas Medical Association at Galveston, May 9th, 10 and 11th.

About seventy-five of the leading surgeons of the State attended the first session of the annual convention of the Texas Surgical Association at the Adolphus Hotel, Dallas, recently.

Recent studies of the modes of attack by tubercle bacilli indicate that children are much more easily infected than adults, and that in most cases the disease is contracted in very early childhood. Practically no child in a family where there is open tuberculosis escapes.

Work is being rushed on the new building being constructed at the Texas Tuberculosis Sanitarium at Carlsbad. It is hoped to have all the buildings completed within the next few months.

---

A dispatch from London says all medical men throughout the country, regardless of age, are being asked to enroll themselves to meet "an urgent national need." The work of enrollment is being conducted by the Medical War Department in England, Scotland and Ireland.

---

Contrary to statements regarding satisfactory food supplies at the front, Dr. W. L. Brown, a physician of El Paso, who returned recently from Casas Grandes, says the advance grades near Parrel are in actual want and that unless more adequate means of communication are arranged they may suffer seriously.

---

The Austrian military authorities have decided to vaccinate, or revaccinate, the whole population of some three and one-half millions of Galicia. As a beginning, six hundred men and women medical students at Cracow University are taking a three weeks' practical course in learning how to carry out this immense work.

---

The heroism of two British air men was demonstrated recently when one of them amputated the fingers of another while under fire. They were being chased by a German aeroplane. The British aeroplane began to descend and the Germans fired, wounding the captain in the right arm and smashing two of his fingers. While the captain steered with his left hand the lieutenant amputated the two fingers.

---

A locomotive engineer who since June, 1915, has been languishing in a hospital bed in Dallas suffering from severe burns received by being scalded with steam when his locomotive was wrecked, physicians say, will recover as a result of skin-grafting operations, one of the most remarkable cases on record. Nearly fifty persons have gone under the surgeon's knife to give patches of skin to be grafted on the engineer's back.

---

J. R. Stratton, of Peterboro, Ontario, Canada, died recently after thirty-six hours of fasting, and his physician, Prof. K. Feigh, formerly of Oklahoma City, has been arrested for manslaughter. Feigh had prohibited Stratton from taking any food, it was testified.

The victim had hiccoughs for seventy-four hours, which Feigh had attempted to cure by binding a strap around his stomach. When local physicians were called in they could not save his life.

---

CHRONIC GASTRITIS.—For years I have prescribed berberine, nux, xanthoxylon or capsicum, and perhaps a little iron carbonate, if the patient is anemic.—*Ellingwood.*

---

### Little Damage to the Abbott Laboratories.

A small fire with explosion of gases occurred April 21st on the top floor of one of the buildings of the Abbott laboratories. Newspaper reports of the extent and character of this accident were grossly exaggerated. The damage was very small, consisting mainly of broken window panes and cracking of temporary partitions. The plant and machinery were injured but slightly, and the entire force went to work the next morning as usual. The Abbott laboratories have issued a statement positively denying the newspaper reports that this firm is or has been engaged in the manufacture of ammunition or explosives.

---

Doctor—"Mr. Blinks, your wife is very ill, indeed."

Blinks—"Let me know the worst at once—is it Atlantic City, Palm Beach or San Francisco?"—*Philadelphia Ledger.*

---

### An Open Letter.

DEAR DOCTOR: We offer you at the Westbrook Hotel much the best and most desirable accommodations in Fort Worth, and the rates are no higher than the others.

Absolutely fireproof; clean snowy white beds, excellent service and the best cooking in Texas at reasonable prices.

Our room rates are: One person, without bath, $1.00 to $1.50; two persons, without bath, $2.00 to $3.00; one person, with bath, $1.50 to $3.00; two persons, with bath, $2.50 to $4.50.

We extend you a cordial invitation to give us a trial when you again visit Fort Worth.

Yours very truly,

WESTBROOK HOTEL CO.,

H. B. Christian, President.

[Don't forget that the Westbrook is the doctors' headquarters when they visit Fort Worth.—ED.

Seventy-six out of eighty-seven cases of typhoid fever which occurred in a recent outbreak have been traced by the United States Public Health Service to infected milk. Had the first cases been reported to a trained health officer the outbreak could have been stamped out promptly. When will we learn that disease prevention is sure and cheap?

---

Judge—"Officer, what's the matter with the prisoner? Tell her to stop that crying—she's been at it fifteen minutes" (more sobs).

Officer—"Please, sir, I'm a-thinking she wants to be bailed out."—*Nebraska Awgwan.*

